# Evaluation of a device to detect neonatal hypothermia in a clinical setting in Ghana

**DOI:** 10.1371/journal.pgph.0001681

**Published:** 2023-10-24

**Authors:** Lauren McAbee, Paddington T. Mundagowa, Babbel Agbinko-Djobalar, Prince Gyebi Owusu, Adziri Sackey, Isabel Sagoe-Moses, Emma Sacks, Kwame Sarfo Sakyi, Robin B. Dail, Mufaro Kanyangarara

**Affiliations:** 1 Department of Epidemiology and Biostatistics, University of South Carolina, Arnold School of Public Health, Columbia, South Carolina, United States of America; 2 Korle-Bu Teaching Hospital, Accra, Ghana; 3 Center for Learning and Childhood Development-Ghana, Accra, Ghana; 4 Michigan State University, East Lansing, Michigan, United States of America; 5 Ghana Health Services, Accra, Ghana; 6 Department of International Health, Johns Hopkins School of Public Health, Baltimore, Maryland, United States of America; 7 Public and Environmental Wellness, School of Health Sciences, Oakland University, Rochester, Michigan, United States of America; 8 University of South Carolina, College of Nursing, Columbia, South Carolina, United States of America; University of Global Health Equity, RWANDA

## Abstract

Neonatal hypothermia poses an increased risk of infection, hypoglycemia, metabolic dysfunction, and mortality, particularly in preterm or low birthweight (LBW) infants. However, early detection of hypothermia and prompt thermoregulation can mitigate these effects thus, the need for continuous neonatal temperature monitoring. The BEMPU TempWatch is a small bracelet designed for continuous temperature monitoring for neonates. When the body temperature falls below 36.5˚C, the bracelet generates an alarm sound and flashes an orange light, indicating hypothermia. This study aimed to assess the validity of the BEMPU TempWatch in detecting hypothermia in a clinical setting in Ghana using sensitivity and specificity. Additionally, the study sought to identify factors associated with misclassification using logistic regression analysis. A standardized questionnaire collected information about the mother, pregnancy, delivery, and neonate. The BEMPU TempWatch was placed on the wrist of the neonate, and over a 24-hour follow-up period, a nurse took 4-hourly axillary temperature readings using a digital thermometer. Whenever the device’s alarm sounded, a nurse immediately checked and recorded the axillary temperature, undertook necessary clinical actions, and rechecked after 30 minutes. Among the 249 neonates included in the study, 57.0% were female, 12.5% were extremely LBW, and 13.7% were extremely preterm. Based on 1,973 temperature readings, the sensitivity of the BEMPU TempWatch in detecting hypothermia was 67.8%, and the specificity was 95.9%. The sensitivity was lower among neonates being treated in incubators (58.4%) compared to those not (82.7%). Sensitivity was higher among neonates with LBW (1,500–2,500g) (73.5%) than very or extremely LBW neonates (<1,500g) (62.8%). The results showed that the BEMPU TempWatch had significantly fewer misclassifications among neonates who were not treated in an incubator, received only breastmilk, and were not born extremely preterm. Further studies are warranted to evaluate the effectiveness of the BEMPU TempWatch on neonatal health outcomes.

## Introduction

In 2021, 5.0 million children under five years of age died, with 2.3 million of those deaths occurring within the first month of life [1]. While there has been a decline in neonatal mortality in recent years, it has been slower compared to the decline in mortality among children 1–59 months of age [1]. Low birth weight (< 2,500 grams at birth) due to preterm birth or intrauterine growth restriction is a major determinant of neonatal mortality. Of the 15 million infants born before 37 completed weeks of gestation, one million die from preterm birth complications [2,3]. Low birth weight (LBW) and preterm infants surviving the neonatal period have an increased risk of infections, gastrointestinal problems, immunological issues, and long-term cognitive and neurodevelopmental impairments compared to term and normal weight infants [4]. Additionally, these vulnerable infants are susceptible to neonatal hypothermia due to relatively high heat loss and diminished ability to produce heat, leading to difficulty maintaining normothermia [5,6]. Neonatal hypothermia occurs when the body temperature falls below 36.5˚C and can be classified as mild (36.0–36.4˚C), moderate (32.0–35.9˚C), or severe (<32˚C) [7]. Neonatal hypothermia is typically accompanied by poor feeding, inadequate weight gain, lethargy, a weak cry, reduced movements, and skin cold to the touch [7,8]. Neonatal hypothermia increases the susceptibility of newborns to severe conditions, including sepsis, pneumonia, respiratory distress syndrome, hypoglycemia, jaundice, sepsis, metabolic acidosis, birth asphyxia, and even death in the absence of preventive or immediate care [7–9]. Although neonatal hypothermia is not considered a direct cause of neonatal death, it substantially contributes to neonatal mortality [10].

Early detection of hypothermia is crucial to promptly implementing interventions to prevent or mitigate the negative consequences linked to neonatal hypothermia. Warming the delivery rooms, drying the infant immediately after birth, initiating skin-to-skin contact, supporting breastfeeding, delaying bathing, provision of appropriate clothing, warm transport within the hospital, keeping the mother and infant together, providing proper thermal care during resuscitation, and improving awareness and recognition of risks associated with hypothermia are recommended to avert hypothermia [7]. Kangaroo Mother Care (KMC), a widely used intervention, consists of skin-to-skin contact, exclusive breastfeeding, early discharge from the hospital, and continuous support for infants and caregivers at home. KMC has demonstrated efficacy in reducing hypothermia, neonatal infections, and neonatal mortality in both facility and community settings, while promoting weight gain and parent-infant bonding [11]. Moreover, universal adoption of KMC for LBW and preterm infants could avert up to 450,000 neonatal deaths annually [12]. Despite its benefits, widespread implementation of KMC has been hindered by structural, economic, logistic, and social barriers [12].

Recent advances in wearable technology have opened up opportunities for the early detection of neonatal hypothermia, facilitating timely clinical action to prevent associated adverse outcomes [13,14]. Current continuous temperature monitoring devices for at-risk neonates in low-resource settings include ThermoSpot, the neonatal temperature monitor (NTM), and the BEMPU TempWatch [14]. Developed by Bempu Health (Bangalore, India), the BEMPU TempWatch provides real-time continuous monitoring of the body temperature of neonates weighing between 800 and 3,300 grams, and can be used for up to four weeks [13–15]. The device comprises a silicone band equipped with an audio alarm and an orange light flash emitting thermistor metal cup, which activates when the body temperature drops below 36.5⁰C, signaling the onset of hypothermia and prompting the provision of thermal care. The device capitalizes on the physiological phenomenon where peripheries cool before the central core temperature drops indicating heat loss and impending hypothermia. A validation study conducted in Pondicherry, India, demonstrated high diagnostic accuracy of the BEMPU TempWatch in detecting neonatal hypothermia among neonates weighing less than 2,000 grams, with a sensitivity of 98.6% and a specificity of 95.0% [16]. Further studies in India suggest that using the BEMPU TempWatch at home lowers mortality risk in the first four weeks of life and improves average daily weight gain [17,18]. In addition, the device may enhance maternal confidence in caring for LBW and preterm infants and lessen the burden associated with their care [18].

The BEMPU TempWatch is marketed in low-resource settings and has already been distributed in Benin, Cameroon, Guinea Bissau, India, Kenya, Nepal, Papua New Guinea, Togo, and Zimbabwe. Despite growing interest in integrating and expanding the use of the device in clinical and community settings, there is still insufficient data to support the routine use of the BEMPU TempWatch in LBW and preterm neonates in low-resource settings. Only two studies in India have examined the accuracy of the BEMPU TempWatch in detecting hypothermia among infants in neonatal intensive care units (NICUs). Given the limited evidence, validation studies in other field settings are needed [19]. The present study aimed to assess the validity of the BEMPU TempWatch in detecting neonatal hypothermia in a clinical setting in Ghana. Notably, the neonatal mortality rate in Ghana is an estimated 22.9 deaths per 1,000 live births, resulting in over 20,000 neonatal deaths annually [1]. The country has a preterm birth rate of 12%, contributing to an estimated 104,926 preterm births every year [20]. Previous studies conducted in tertiary hospitals in Ghana found that upon admission to the NICU, 59–68% of infants were hypothermic [21–23]. Taken together, these statistics highlight the high burden of neonatal hypothermia and underscore the need for interventions such as the BEMPU TempWatch to prevent and treat hypothermia.

## Methods

### Study design and setting

A prospective controlled study design was employed for this study. Participants were recruited from the Korle-Bu Teaching Hospital in Accra, Ghana from May 2021 to January 2022. The hospital has a capacity of over 2,000 beds and its NICU has 60 cots, warming platforms, and incubators. Approximately 80% of NICU admissions are referrals from Korle-Bu Teaching Hospital labor wards and obstetric theater, while the remaining admissions come from health facilities in the southern part of Ghana [24]. The average length of stay for neonates in the NICU is about 14 days. Neonates admitted to the NICU, who were <28 days old, had a birth weight <2,500 grams (regardless of gestational age), were clinically stable, and whose mother was available and willing to provide consent were eligible to participate. Clinical stability was defined as breathing spontaneously without requiring additional oxygen. Neonates with an anticipated discharge in less than 24 hours, those who were clinically unstable, or those whose mothers were below 18 years old were excluded from the study.

### Sample size

The minimum sample size required to estimate the sensitivity and specificity of the BEMPU TempWatch was determined using Buderer’s formula, assuming an alpha of 0.05, a desired precision of 0.05, and z1−α22 = 3.84 [25]. The prevalence of hypothermia was based on a previous study in a tertiary hospital in eastern Ghana, which found that 59% of neonates in the NICU had an axillary temperature below 36.5°C on admission [23]. The expected sensitivity and specificity of the BEMPU TempWatch in detecting hypothermia were based on the India study that validated the device among 461 hospitalized neonates (sensitivity 98.6% and specificity 95.0%) [16]. Accounting for possible loss to follow-up and missing data, the minimum required sample size was determined to be 243 neonates.

### Study procedures

Neonates admitted to the NICU at Korle-Bu Teaching Hospital were screened for eligibility to participate in the study. As our focus was on LBW infants at risk for hypothermia, we did not recruit from the labor and delivery ward and KMC ward; instead, we exclusively recruited from the NICU, where more than half of admissions are LBW infants [24]. Once written informed consent to participate in the study was obtained from the mother, a trained study nurse administered a standardized questionnaire to collect data on sociodemographic characteristics. Subsequently, the study nurse applied the BEMPU TempWatch to the neonate’s wrist, after which the neonate was returned to their cot or incubator if receiving thermal care. Axillary temperature readings were taken using a digital thermometer every four hours for 24 consecutive hours. At each time point, the study nurse ensured the device was correctly worn, recorded its status, and monitored functionality. During the 24-hour period, whenever the device emitted an alarm sound indicating hypothermia, the study nurse immediately checked and recorded the neonate’s axillary temperature and the clinical actions taken, if any. Clinical actions included transferring the neonate to an incubator, adjusting the temperature of the incubator, wrapping the neonate, adding clothing layers, and initiating skin-to-skin contact. The temperature of the neonate was taken again 30 minutes after the alarm to monitor progress. All data on the temperatures, the status of the BEMPU TempWatch, and thermal care practices were recorded on a standardized temperature monitoring form. Data were entered into an online form using Kobo Toolbox (www.kobotoolbox.org).

### Statistical analysis

To assess the validity of the BEMPU TempWatch in detecting hypothermia, the sensitivity and specificity were estimated by comparing the status of the device (orange indicator or alarm) to the axillary temperature measured at each time point. Axillary temperature was selected as the reference as it is routinely used to monitor neonatal temperatures in this setting. Specificity was defined as the proportion of occasions when the axillary temperature reading was not below 36.5˚C and the device did not indicate hypothermia (true negatives). Sensitivity was defined as the proportion of occasions when the axillary temperature reading was below 36.5˚C, and the device indicated hypothermia (true positives). Positive and negative predictive values, accuracy, and area under the receiver operating characteristic (ROC) curve were also computed. The discriminative ability of the BEMPU TempWatch in correctly identifying hypothermia was assessed using the following classification of the area under the curve (AUC): <0.5 indicated no discrimination, 0.7–0.8 indicated acceptable discrimination, 0.8–0.9 indicated excellent discrimination, and >0.9 indicated outstanding discrimination [26,27]. The study also investigated whether the device’s validity varied by birth weight and incubator use.

The study also examined whether specific clinical and practice factors were associated with misclassifications in the BEMPU TempWatch–i.e., false positives and false negatives. False positives represented occasions when the device generated an alarm or flashed orange, indicating hypothermia (<36.5˚C), but the digital thermometer reading indicated an axillary temperature at or above 36.5˚C. False negatives represented occasions when the temperature of the neonate was below 36.5˚C, yet the device did not generate an alarm or flash orange to indicate hypothermia. Factors of interest included gestational age, birth weight, mode of delivery, whether the neonate was being treated in an incubator, room temperature, thermal care practices at the time of the temperature reading (i.e., wearing socks, a diaper, a cap, or mittens) and feeding practices (breastmilk, formula, both or other). Gestational age was calculated using the last menstrual period or early ultrasound scan if available. Room temperature (hot, cold, normal) was based on the report of the study nurse. Univariate and multivariate logistic regression analyses were performed to identify factors associated with false positives and false negatives, respectively. The multivariate model used a backward stepwise procedure to retain statistically significant variables (p<0.05). Unadjusted and adjusted odds ratios (OR) and 95% confidence intervals (CI) were presented as measures of association. Correlations from repeated temperature readings on the same neonates were accounted for using generalized estimating equations (GEE). STATA BE/17.0 (College Station, Texas) was used to perform all statistical analyses. A p-value <0.05 was considered statistically significant.

### Ethical considerations

Ethical approval was obtained from the Institutional Review Boards at the University of South Carolina (Protocol 00095600) and Korle Bu Teaching Hospital (KBTH-IRB/00052/2020) before any interaction with human subjects. The use of the BEMPU TempWatch in a clinical setting was approved by the Ghana Food and Drugs Authority (FDA) (FDA/D.21-3138). Written informed consent was obtained from each mother. Several measures, including wearing face masks, handwashing and sanitizing hands before procedures, and social distancing, were implemented to ensure the safe conduct of research during the COVID-19 pandemic.

Additional information regarding the ethical, cultural, and scientific considerations specific to inclusivity in global research is included in the (S1 Text).

## Results

Between May 2021 and January 2022, a total of 255 neonates were enrolled in the study. Six of them were excluded from analysis due to missing data. Of the 249 included neonates, more than half were female (57.0%), fed only breastmilk (67.1%), delivered via cesarean section (53.4%), and not being treated in an incubator at the time of enrolment (68.4%) (Table 1). All neonates in the study had a birth weight <2,500 grams, with 36.1% classified as very low birth weight (1,000–1,499 grams) and 12.5% as extremely low birth weight (<1,000 grams). In terms of gestational age at birth, 40.6% of neonates were born moderate-late preterm (32–36 weeks), 40.6% were born very preterm (28–31 weeks), 13.7% were born extremely preterm (<28 weeks); and only 5.2% were born full-term (37–40 weeks). Almost half (47%) of neonates were less than 7 days at enrollment.

**Table 1:** Characteristics of participating neonates (n = 249).

Characteristic	Total Population, n (%)
Sex	
Male	107 (43.0)
Female	142 (57.0)
Birth weight	
Low birth weight (1,500–2,500 g)	128 (51.4)
Very low birth weight (1,000–1,499 g)	90 (36.1)
Extremely low birth weight (<1,000 g)	31 (12.5)
Current feeding practices	
Breastmilk	167 (67.1)
Formula	7 (2.8)
Both	10 (4.0)
Other	65 (26.1)
Method of delivery	
Vaginal	116 (46.6)
C-section	133 (53.4)
Neonate being treated in incubator	78 (31.6)
Gestational age	
Term (37–40 weeks)	13 (5.2)
Moderate-late preterm (32–36 weeks)	101 (40.6)
Very preterm (28–31 weeks)	101 (40.6)
Extremely preterm (Less than 28 weeks)	34 (13.7)
Age at enrolment	
<7 days	117 (47.0)
7–13 days	61 (24.5)
14–20 days	37 (14.9)
21–27 days	34 (13.7)

A total of 1,973 temperature readings were collected and recorded (Table 2). Overall, the BEMPU TempWatch demonstrated an accuracy of 90.6%. The sensitivity of the device in detecting hypothermia was 67.8%, and the specificity in identifying non-hypothermic temperatures was 95.9%. The positive and negative predictive values were 79.1% and 92.8%, respectively. A plot of sensitivity against 1-specificity showed an area under the receiver operating characteristic curve of 0.81, indicating excellent discrimination.

**Table 2:** Sensitivity, specificity, positive predictive value (PPV), negative predictive value (NPV), and accuracy of the BEMPU TempWatch.

	Sensitivity (%)	Specificity (%)	PPV (%)	NPV (%)	Accuracy (%)
All neonates	250/369 (67.8%)	1,538/1,604 (95.9%)	250/316 (79.1%)	1,538/1,657 (92.8%)	90.6%
Incubator	87/149 (58.4%)	1,039/1,064 (97.7%)	87/112 (77.7%)	1,039/1,101 (94.4%)	92.8%
Not in incubators	139/168 (82.7%)	433/470 (92.1%)	139/176 (79.0%)	433/462 (93.7%)	89.7%
Low birth weight (1,500–2,500 g)	125/170 (73.5%)	810/848 (95.5%)	125/163 (76.7%)	810/855 (94.7%)	91.8%
Very or extremely low birth weight (<1,500 g)	125/199 (62.8%)	728/756 (96.3%)	125/153 (81.7%)	728/802 (90.8%)	89.3%

### Factors associated with false positives

Of the 1,973 temperature readings, 66 (3.3%) were false positives. In the univariate analysis, neonates treated in an incubator had almost three times odds of a false positive than those not treated in an incubator (OR 2.86 95%, CI 1.71–4.81) (Table 3). Neonates fed with formula also had increased odds of a false positive (OR 2.60, 95% CI 0.99–6.85). Factors that were associated with decreased odds of a false positive were being wrapped in a blanket or cloth (OR 0.45, 95% CI 0.27–0.74) and being born very preterm (OR 0.37, 95% CI 0.13–1.04) or extremely preterm (OR 0.31, 95% CI 0.08–1.17). Multivariate results indicated that neonates treated in an incubator were almost three times more likely to experience a false positive than those not treated in an incubator (aOR 2.72, 95% CI 1.59–4.63). Neonates fed formula had increased odds of a false positive compared to those fed only breastmilk (aOR 3.49, 95% CI 1.29–9.48).

**Table 3:** Univariate and multivariate logistic regression for factors associated with false positives (n-1,697).

Factors	Univariate			Multivariate		
	OR	95% CI	p-value	aOR	95% CI	p-value
Neonate being treated in incubator	2.86	1.71–4.81	<0.001	2.72	1.59–4.63	<0.001
**Birth weight**						
Low birth weight (1,500–2,499g)	Ref					
Very low birth weight (1,000–1,499g)	0.64	0.35–1.16	0.1			
Extremely low birth weight (<1,000g)	1.43	0.75–2.74	0.3			
**Method of delivery**						
Vaginal	Ref					
C-section	0.90	0.55–1.48	0.7			
**Current feeding practices**						
Breastmilk	Ref			Ref		
Formula	2.60	0.99–6.85	0.05	3.49	1.29–9.48	0.01
Both	0.69	0.16–2.89	0.6	0.50	0.12–2.11	0.3
Other	0.48	0.23–0.98	0.05	0.64	0.30–1.33	0.2
**Neonate clothing**						
Wearing socks	2.40	0.33–17.60	0.4			
Wearing cap/hat	1.12	0.56–2.24	0.7			
Wrapped with blanket/cloth	0.45	0.27–0.74	0.002			
**Room temperature**						
Hot	Ref					
Cold	1.76	0.15–20.40	0.7			
Normal	1.10	0.15–8.21	0.9			
**Gestational age**						
Full-term (37–40 weeks)	Ref					
Moderate-late preterm (32–36 weeks)	0.99	0.38–2.56	1.0			
Very preterm (28–31 weeks)	0.37	0.13–1.04	0.06			
Extremely preterm (< 28 weeks)	0.31	0.08–1.17	0.08			

OR: Odds ratio, aOR: Adjusted odds ratio, CI: Confidence interval, Ref: Reference.

### Factors associated with false negatives

Of the 1,973 temperature readings, 119 (6.0%) were false negatives. Based on the univariate results, neonates born with a very low birth weight (1,000–1,499 grams) (OR 1.58, 95% CI 1.04–2.40) or an extremely low birth weight (<1,000 grams) (OR 2.45, 95% CI 1.49–4.04) had increased odds of a false negative when compared to those with a low birth weight (1,500–2,499 grams) (Table 4). Neonates fed both breastmilk and formula had increased odds of a false negative (OR 2.50, 95% CI 1.23–5.06). Compared to neonates born full-term, those born extremely preterm had a significantly higher likelihood of a false negative (OR 12.64, 95% CI 1.70–93.84). Wearing a cap/hat (OR 1.80, 95% CI 0.92–3.52) or a diaper (OR 3.25, 95% CI 0.93–11.29) was associated with increased odds of a false negative. Multivariate results showed that neonates fed both breastmilk and formula were more than three times as likely to have a false negative compared to those fed only breastmilk (aOR 3.41, 95% CI 1.65–7.04). Neonates born extremely preterm had a significantly higher likelihood of a false negative than neonates born full-term (aOR 15.93, 05% CI 2.10–120.81).

**Table 4:** Univariate and multivariate logistic regression for factors associated with false negatives (n = 1,845).

Factors	Univariate			Multivariate		
	OR	95% CI	p-value	aOR	95% CI	p-value
Neonate being treated in incubator	0.89	0.57–1.40	0.6			
**Birth weight**						
Low birth weight (1,500–2,499g)	Ref					
Very low birth weight (1,000–1,499g)	1.58	1.04–2.40	0.03			
Extremely low birth weight (<1,000g)	2.45	1.49–4.04	<0.001			
**Method of delivery**						
Vaginal	Ref					
C-section	0.70	0.48–1.01	0.06			
**Current feeding practices**						
Breastmilk	Ref			Ref		
Formula	0.75	0.18–3.18	0.7	0.65	0.15–2.81	0.6
Both	2.50	1.23–5.06	0.01	3.41	1.65–7.04	0.001
Other	1.51	1.00–2.26	0.05	1.40	0.92–2.12	0.1
**Neonate clothing**						
Wearing socks	3.70	0.51–26.99	0.2			
Wearing cap/hat	1.80	0.92–3.52	0.08			
Wrapped with blanket/cloth	3.25	0.93–11.29	0.06			
**Room temperature**						
Hot	Ref					
Cold	0.88	0.05–14.69	0.9			
Normal	2.09	0.28–15.51	0.5			
**Gestational age**						
Full-term (37–40 weeks)	Ref			Ref		
Moderate-late preterm (32–36 weeks)	5.05	0.69–37.13	0.1	6.05	0.81–45.00	0.08
Very preterm (28–31 weeks)	5.27	0.72–38.74	0.1	6.11	0.82–45.62	0.08
Extremely preterm (< 28 weeks)	12.64	1.70–93.84	0.01	15.93	2.10–120.81	0.007

OR: Odds ratio, aOR: Adjusted odds ratio, CI: Confidence interval, Ref: Reference.

## Discussion

This study aimed to assess the validity of the BEMPU TempWatch in detecting neonatal hypothermia among neonates in a clinical setting in Ghana. The study also identified factors associated with misclassification of hypothermia by the device (i.e., false positives and negatives). The device demonstrated high specificity (95.9%) in accurately detecting non-hypothermic temperatures and moderate sensitivity (67.8%) in accurately detecting hypothermic temperatures (<36.5°C). It is noteworthy that the previous validation study of the BEMPU TempWatch in India demonstrated high sensitivity (98.6%) and specificity (95.0%) [16]. While the specificity in the present study is similarly high, the sensitivity is lower (67.8%) than in the validation study in India. The difference in sensitivity could potentially be attributed to variations in the study populations, as the India study enrolled neonates with a birth weight of less than 2,000 grams who were not receiving incubator care [16]. By contrast, our study enrolled neonates with a birth weight of less than 2,500 grams, irrespective of incubator use. Notably, when analyzing temperature readings for neonates not in incubators, the sensitivity increased to 82.7%, which is more aligned with the India study. It is also worth noting that 67% of the neonates in the India study exhibited hypothermia during the 24-hour monitoring period [16], compared to 45% in our study. Arguably, the sensitivity of the device is more important than the specificity, as a high sensitivity in detecting hypothermia helps healthcare providers intervene early before hypothermia deteriorates into a severe condition. In our study, the device demonstrated inconsistency in detecting hypothermia among neonates who were hypothermic and very or extremely low birth weight or were already receiving thermal care in an incubator.

Consistent with the previous study in India, our study also found that the BEMPU TempWatch had high positive and negative predictive values of 79.1% and 92.8%, respectively. The high diagnostic accuracy of the device indicated there is an 81% chance the device will correctly distinguish a non-hypothermic temperature from a hypothermic temperature. The India study similarly reported high positive and negative predictive values of 83.6% and 99.6%, respectively [16]. Our positive results reflect consistency in the device’s ability to detect neonatal hypothermia.

Misclassifications that do not correctly identify hypothermic neonates are of most concern, as these vulnerable neonates require immediate attention. Although false positives might be acceptable if healthcare workers understand the low threshold for screening, frequent inaccuracies may erode trust in the device, leading to alerts being ignored. Our study found that the device was more accurate among neonates who were not born extremely preterm, were exclusively fed breastmilk, and were not being treated in an incubator. Multivariate results showed that extremely preterm infants were more likely to experience false negatives than other neonates. While the size of the device is adjustable, the device may fit loosely on the smaller wrists of extremely preterm neonates, resulting in inaccurate temperature readings and misclassifications. Therefore, for extremely preterm neonates, the device’s suitability might be challenged, especially considering the standard of care is a thermistor.

Interestingly, our study also showed that misclassifications by the BEMPU TempWatch were more likely among neonates who fed formula or a combination of formula and breastmilk compared to those exclusively breastfed. While exclusive breastfeeding offers numerous benefits, including preventing and treating neonatal hypothermia, there is no clear explanation for this association. Additionally, given the small sample sizes of formula-fed (n = 7) and mixed-fed (n = 10) neonates, caution should be used in interpreting this finding. We also found that misclassifications were more likely among neonates being treated in an incubator. Incubators primarily prevent or treat hypothermia by enclosing neonates in a small, warm, temperature-controlled environment. Fluctuations in ambient temperature in an incubator might influence peripheral wrist temperatures, resulting in significant differences between wrist and axillary temperatures. Several studies have documented differences in temperature measurements across various body sites [28,29]. Qualitative interviews with clinicians at Korle-Bu Teaching Hospital suggested that within the noisy NICU environment, the audibility of the device’s alert for infants in incubators might be compromised, possibly impacting the accuracy of the device [30]. Moreover, interviewed clinicians advocated for incorporating a digital display of the actual temperature to enhance the usefulness of the device in clinical settings.

The study had a few limitations worth noting. Firstly, it was only conducted in one teaching hospital with a high patient volume in Ghana, and therefore, the results may not be generalizable to other settings. The study focused on a select set of neonate characteristics, and other factors affecting the accuracy of the BEMPU TempWatch might have been overlooked. Additionally, factors such as room temperature and last menstrual period (used to estimate gestational age) were based on self-report, potentially introducing reporting errors. Temperature data from both the NICU room and incubators would have enhanced our contextual understanding. Lastly, the BEMPU TempWatch detects hypothermia using peripheral wrist temperatures, which may differ from axillary temperatures, especially given the wide range of birth weights in our study. Future research could consider modifications to the device to track temperatures on the upper arm instead of the wrist, potentially aligning more closely with core body temperature.

Despite these limitations, the study provided encouraging results regarding the sensitivity, specificity, and overall accuracy of the device. The clinical performance of the BEMPU TempWatch was characterized by high specificity, moderate sensitivity, and excellent diagnostic accuracy. Our study also highlighted factors influencing the accuracy of the device. Specifically, we found that the device may not work as well among extremely preterm neonates, very LBW neonates, and neonates receiving care in NICU incubators, suggesting the need for further evaluation in other settings and possible adjustments to enhance the accuracy in these populations. Well-designed randomized controlled trials investigating the impact of the device on hypothermia and neonatal mortality, with a focus on populations for whom the device is most accurate, will be essential before scaling up the use of the device. Hypothermia is often missed during routine four hourly temperature checks. Although the BEMPU TempWatch may be less suited for high-resource settings [31], its ability to continuously monitor temperature and accurately detect neonatal hypothermia early between intermittent temperature checks could be advantageous in low-resource settings with fewer healthcare workers and limited resources.

## Supporting information

S1 Data Data supporting the findings. (XLSX)

S1 Text Inclusivity in global research. (DOCX)
